# Examining the Impact of Skin Lighteners *In Vitro*

**DOI:** 10.1155/2013/702120

**Published:** 2013-04-28

**Authors:** James V. Gruber, Robert Holtz

**Affiliations:** ^1^Lonza Consumer Care, 70 Tyler Place, South Plainfield, NJ 07080, USA; ^2^BioInnovation Laboratories, Inc., 7220 W. Jefferson Avenue STE 112, Lakewood, CO 80235, USA

## Abstract

Three cosmetically important skin lightening agents, hydroquinone (HQ), kojic acid (KA), and niacinamide (NA), consume the bulk of successful skin lightening ingredients in cosmetic applications. However, the mechanisms by which these ingredients work are still unclear. In this study, melanocytes and keratinocytes were treated with high, nontoxic doses of HQ, KA, and NA and the cells were examined by human microarrays and protein assays for several important targets including cytotoxicity, melanin expression, tyrosinase gene (TYR) and protein expression, melanocortin-1 receptor (MC1R) gene and protein expression, cytochrome c oxidase-1 (COX1) gene and protein expression, and ferritin (FTH1) gene and protein expression. It was found that all the skin lighteners examined showed marked increases in TYR, COX1, and FTH1 gene and protein expression, but not in MC1R expression in melanocytes. Upregulation of COX1 and FTH1 genes and proteins was common across both cell lines, melanocytes and keratinocytes. The results of the tyrosinase expression were somewhat unexpected. The role of iron in the expression of melanin is somewhat unexplored, but common and strong upregulation of ferritin protein in both types of cells due to the treatments suggests that iron plays a more pivotal role in melanin synthesis than previously anticipated.

## 1. Introduction

Mechanisms of melanogenesis are complex and a number of recent publications have summarized the current understanding of the process of melanin production in the skin [1–5]. It is well understood that the rate controlling enzyme for melanin formation is tyrosinase (TYR) which is responsible for several oxidative steps in the synthesis of melanin [6, 7]. Melanocortin-1 receptor (MC1R) is responsible for binding melanocyte stimulating hormone (MSH), expressed by stressed keratinocytes, and initiating the cascade of melanogenesis [8]. The role of cytochrome c oxidase in melanin synthesis has been suggested to principally occur through the cytochrome c/H_2_O_2_ oxidation of catecholamines [9]. Likewise, the role of iron in the skin pigmentation process is also somewhat vague. Perhaps the best work in this area was conducted by Palumbo et al. who demonstrated that ferrous ions can help drive tyrosinase activity in melanocytes [10, 11].

In this work, melanocytes were treated with three well established skin lighteners, hydroquinone (HQ), kojic acid (KA), and niacinamide (NA) [12–15]. The highest dosage of each of the ingredients that was noncytotoxic was established using the MTT assay. This established the levels of treatment on normal melanocytes used in the DNA microarray analyses. While multiple genes were influenced by the skin lightening ingredients, four genes of particular interest were chosen to be examined in greater detail because the genomic assays results presented unusual or unanticipated responses. These genes transcribe several proteins which would not have been anticipated to be upregulated by these skin lighteners including tyrosinase (TYR), cytochrome c oxidase (COX1) (not to be confused with the protein cyclooxygenase-1 identified as COX-1), melanocortin-1 receptor protein (MC1R) and ferritin (FTH1). To further elucidate what might be happening to these four cellular markers, melanocytes were further tested with these ingredients at the same noncytotoxic concentrations and protein expression was examined using immunoblot assays. In addition, the cells were examined for their ability to express melanin. The studies were further extended by examining the genomic and protein impact of the same skin lighteners on normal human epidermal keratinocytes (NHEK) examining two of the key markers including COX1 and FTH1. 

## 2. Methods and Materials

Samples of hydroquinone, kojic acid, and niacinamide were purchased from Sigma Chemical and were prepared directly in cell culture media.

### 2.1. Melanocyte Cell Culture

Human epidermal melanocytes were obtained from Cascade Biologics (obtained from a single darkly pigmented donor) and grown in M254 media (supplemented with bovine pituitary extract (BPE) (0.2% v/v), fetal bovine serum (0.5% v/v), insulin (5 *μ*g/mL), transferrin (5 *μ*g/mL), basic fibroblast growth factor (3 ng/mL), hydrocortisone (0.18 *μ*g/mL), heparin (3 *μ*g/mL), phorbol 12-myristate 13-acetate (PMA) (10 ng/mL), and l-tyrosine (0.2 mM)). The cells were seeded into T-25 flasks for the array work, or in well plates for the cytotoxicity, melanin, and protein expression assays, and grown at 37 ± 2°C and 5 ± 1% CO_2_ until confluency. Upon reaching confluency the cells were treated with the various test materials for 24 hours for the array work and the cytotoxicity assay and for 48 and 96 hours for the melanin and protein expression assays.

### 2.2. Keratinocyte Cell Culture

Human epidermal keratinocytes were obtained from Cascade Biologics and grown in Epilife media (supplemented as per the manufacturer's recommendation). For the DNA microarray work the cells were seeded into T-25 flasks, while for the protein expression work the cells were seeded in 24-well plates. In both cases the keratinocytes were grown at 37 ± 2°C and 5 ± 1% CO_2_ until confluency and then treated with the test materials. The treatment time was 24 hours for the array work while 48 hours were used for the protein expression work.

### 2.3. DNA Microarray

After the 24-hour treatment, total RNA was isolated using an RNAqueous Kit (Ambion) as per the manufacturer's instructions. After purification, the total RNA was prepared for array use by first amplifying the RNA using a MessageAmp aRNA Kit (Ambion) and then fluorescently labeling the aRNA with Cy3 or Cy5 using an ASAP Labeling Kit (Perkin Elmer), both as per the manufacturer's instructions. To purify the fluorescently labeled aRNA, a microcon M-30 filter column was inserted into a collection tube and filled with 400 *μ*L of TE buffer. The Cy3 and Cy5 probes were combined and then added to the microcon filter and thoroughly mixed with the TE buffer. The filter was centrifuged at 12,000 RPM for 8 minutes and the flow-through was discarded. The column was then washed twice with 400 *μ*L of TE buffer, discarding the flow-through each time. After the final wash the filter column was inverted, placed into a new collection tube, and centrifuged at 2,000 RPM for 2 minutes to collect the probe. 

The fluorescently labeled aRNA was applied to the DNA microarray chips (Agilent Technologies) and the chip was hybridized overnight and washed as per the manufacturer's recommended protocol. After washing the microarrays were scanned with an Axon GenePix 4100A Scanner with the scanning resolution set to 5 *μ*m and analyzed with GenePix Pro software. During the initial scan the PMT gains for the scanner were adjusted such that the cy5/cy3 image count ratios are between 0.88 and 1.12.

Fluorescence intensities for the microarrays were subjected to global normalization. The total fluorescent signal for both dyes was normalized with a correction factor that would make the ratio of total intensities for both dyes equal to one. For this study a Cy5/Cy3 (treated/untreated) fluorescence intensity ratio greater than 1.3 or less than 0.7 (this relates to a change in gene expression of at least ±30%) was used as the cutoff for- up and down-regulated genes, respectively. 

### 2.4. Melanin Assay

On the final day of treatment the cells used for the melanin assay were washed with PBS and lysed with 100 *μ*L of 1 N NaOH. The well plate was gently rocked to ensure that the 1 N NaOH covered the entire well and complete cell lysis was confirmed via microscopic examination. After the cells were lysed, 100 *μ*L of ultrapure water was added to each well to reduce the concentration of NaOH to 0.5 N. After mixing, 150 *μ*L of each cell lysate was transferred to a 96-well plate. In addition, 150 *μ*L of melanin standards (synthetic melanin obtained from Sigma Chemical, prepared in 0.5 N NaOH) was also transferred to the 96-well plate (in duplicate). The well plate was read at 405 nm using a plate reader. After the melanin assay a 10 *μ*L aliquot of each sample was used to determine the protein concentration of the melanocyte lysate via a BCA Protein Assay.

### 2.5. Melanocyte Lysates: Protein Expression Assays

On the final day of treatment the cells used for the protein expression assay were washed with PBS and then lysed in 200 *μ*L of lysis buffer (1 mM EDTA, 0.5% Triton X-100, 10 mM NaF, 150 mM NaCL, 20 mM *β*-glycerophosphate, 1 mM DTT, 10 *μ*g/mL leupeptin, 10 *μ*g/mL pepstatin, and 3 *μ*g/mL aprotinin prepared in phosphate-buffered saline) on ice for 15 minutes. The protein concentration of the melanocyte lysates was then determined via a BCA Protein Assay. 

### 2.6. Bicinchoninic Acid (BCA) Protein Assay

Fifty volumes of Reagent A (BCA solution) was combined with 1 volume of Reagent B (4% (w/v) CuSO_4_–5H_2_O) in a 15 mL centrifuge tube. Two hundred microliters of this combined reagent was then dispensed into a 96-well plate. Next, 10 *μ*L of each of the standards (bovine serum albumin) or cell lysate sample was added to respective wells. The plate was then covered and incubated at 37 ± 2°C for 30 ± 5 minutes and then read at 540 nm using a microplate reader.

### 2.7. Tyrosinase, MC1R, COX1, and Ferritin Expression: Microfiltration Blotting of Cell Lysate and Immunodetection

A membrane was equilibrated in Tris Buffered Saline (TBS: 20 mM Tris, pH 7.5, 150 mM NaCl) and assembled into a Bio-Dot microfiltration apparatus. After assembly, 200 *μ*L of TBS was added to each well used in the Bio-Dot and the vacuum was applied to ensure that there was adequate flow through all of the wells. Next, each cell lysate sample (approximately 5 *μ*g) was assigned a well in the apparatus and was applied to the appropriate well. The samples were filtered under low vacuum. TBS was added to wells not assigned a sample to ensure that the membrane did not dry out during the procedure. At the end of the blotting procedure an additional 200 *μ*L was applied and filtered through each well. The membrane was then removed from the Bio-Dot apparatus, washed in TBS for 5–10 minutes and then placed into blocking solution (Tris Buffered Saline (20 mM Tris, pH 7.5, 150 mM NaCl, 1% nonfat milk powder)) and allowed to incubate for at least 1 hour at room temperature on a rocking platform.

### 2.8. Antibody Incubation and Detection

After blocking, the membrane was transferred to 20 mL of TBST (TBS with 0.1% Tween-20) and 0.1% nonfat powdered milk with the appropriate primary antibody (all antibodies were obtained from Santa Cruz Biotechnology, Inc., and used at a 1 : 1000 dilution) was added and allowed to incubate overnight at 4°C on a rocking platform. After this incubation the membrane was washed 3 times (1x for 15 minutes and 2x for 5 minutes) in TBST. A fluorescently conjugated secondary antibody (diluted 1 : 2500) was then incubated with the membrane in 15 mL of TBST with 0.1% nonfat powdered milk for 1 hour at room temperature and then washed 3 times with TBS (1x for 15 minutes, and 2x for 5 minutes).

After the final wash, the membrane was placed into a Bio-Rad Molecular Imager FX and scanned using an excitation laser and emission filter combination appropriate for the fluorophore. Images produced by the scanner were then analyzed using ImageJ image analysis software.

## 3. Calculations

### 3.1. Melanin and Protein Assays

The mean absorbance values for the standards were calculated and then used with the standard concentrations to generate an equation for a standard curve using regression analysis. This equation was then used to determine melanin and protein concentrations in the cell lysate samples. 

### 3.2. Image Analysis

Fluorescence intensity measurements were expressed in relative fluorescence units (RFUs). RFUs for the target protein of interest were then normalized to GAPDH. Mean normalized RFU values for each treatment were then calculated and treatments were compared using a one-way ANOVA. 

## 4. Results and Discussion

**Figure 1:**
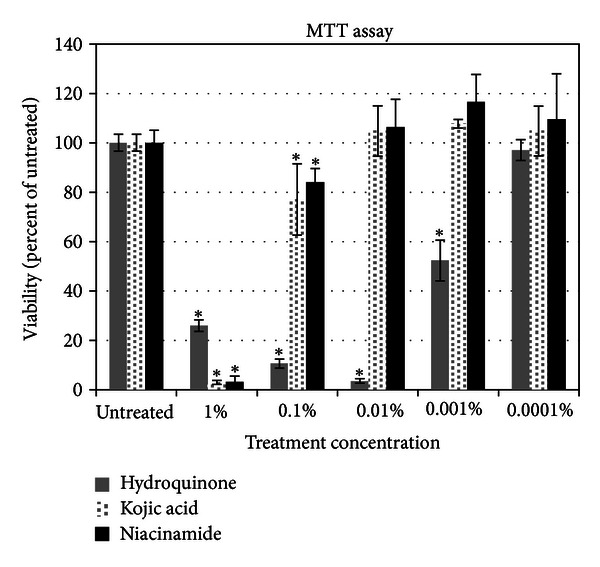
Cell viability assays (MTT) of three skin lightening ingredients on melanocytes.

Results from the MTT assays are shown in Figure 1. It was found that while hydroquinone was nontoxic at 0.0001% when used for a 24-hour incubation in the gene array studies, this concentration was not tolerated for longer incubation periods used for the protein expression assays. Therefore, hydroquinone was also tested in protein assays at 0.00001% and at 0.000001% in the ferritin assays. The shorter timeframe for genomic testing (24 hrs) versus protein assays (48–96 hours) allows for higher concentrations to be tested in the genomic assays without cytotoxic results.

Results from the human microarrays for the four genes mentioned previously are shown in Table 1 showing ratio of medians for each ingredient after 24 hours at the highest noncytotoxic dosages possible (dosages are shown next to each ingredient). It was found that all three skin lighteners upregulated TYR, COX1, and FTH1, but not MC1R. All three skin lighteners appear to have little effect on MC1R expression at the genomic level within the 24-hour timeframe of the arrays and hydroquinone may slightly suppress MC1R gene expression in the 24-hour timeframe. The genes for COX1 and FTH1, however, were quite strongly upregulated in these assays by all three skin lighteners.

**Figure 2:**
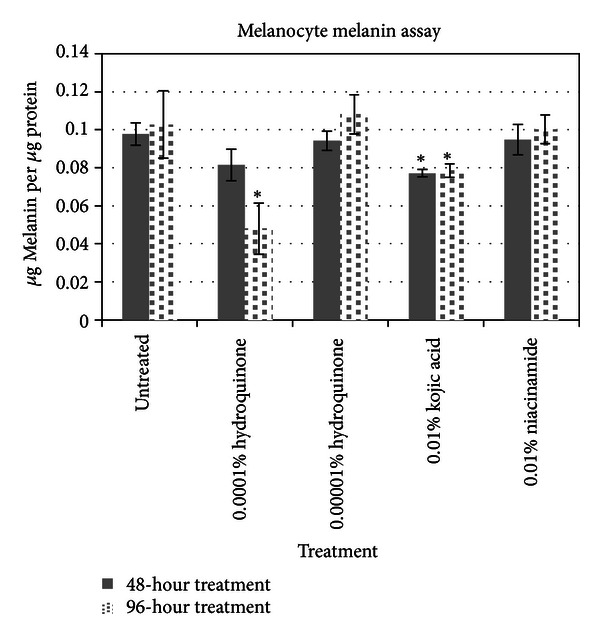
Results of melanin assay results looking at three skin lighteners.

Results from the melanin assays are shown in Figure 2. It appears that while HQ and KA both significantly suppressed melanin expressions, NA did not. However, this is consistent with results shown by Boissy and Hakozaki et al. in similar models [14, 15]. Niacinamide does not appear to suppress direct melanin expression but has been suggested to instead slow transfer of melanin from the melanocytes to the keratinocytes. This effect cannot be detected in melanocyte cell cultures. 

**Figure 3:**
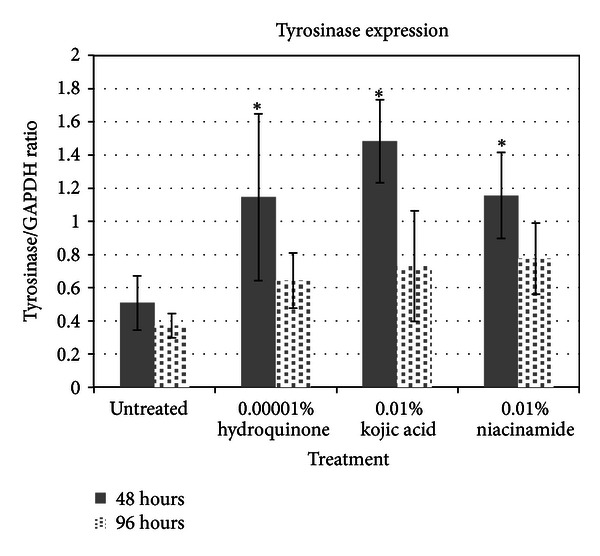
Results from tyrosinase protein expression assays for three skin lighteners.

Results from the expression of tyrosinase are shown in Figure 3. It appears that all three ingredients seem to upregulate tyrosinase expression consistent with the results from the arrays. The upregulation of tyrosinase was somewhat unexpected and is difficult to correlate to products that are supposed to be skin lightening. However, the high levels of tyrosinase in the melanocytes after treatment with skin lightening agents might explain why many people experience hyperpigmentary reactions after ceasing use of skin lightening agents. 

**Figure 4:**
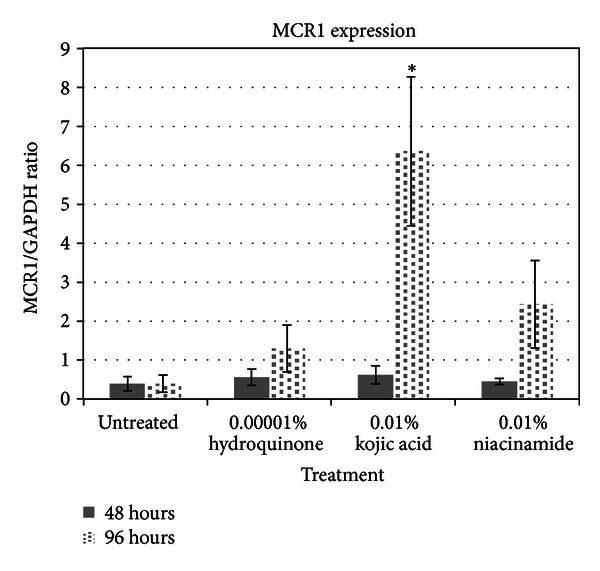
Melanocortin-1 receptor protein expression assay results.

MC1R protein expression (Figure 4) was increased by all three skin lighteners, but the effect did not become apparent until 96 hours into the assay. At 48 hours, MC1R was not upregulated in melanocytes by any of the skin lighteners, consistent with the results from the genomic microarrays.

**Figure 5:**
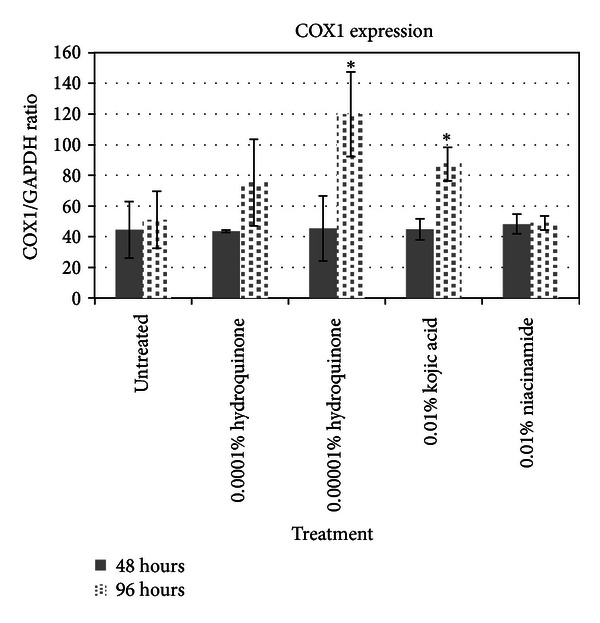
Cytochrome c oxidase-1 protein expression assay results.

Of most particular interest were the results from the COX1 (Figure 5). HQ and KA both had very strong upregulating effects on expression of cytochrome c oxidase, consistent with the genomic data. However, the upregulation of COX1 does not become statistically significant until 96 hours of treatment. It suggests that while gene expression can be noted relatively early, resulting protein expression appears to be delayed by many hours. However, NA, while showing a strong genomic effect, did not increase COX1 protein expression at any time. Thus, the one ingredient that did not show direct melanin suppression in melanocytes also did not show direct upregulation of COX1 protein expression. Whether there is a correlation between COX1 and the ability for melanocytes to move melanin to the keratinocytes is something that would need to be confirmed.

**Figure 6:**
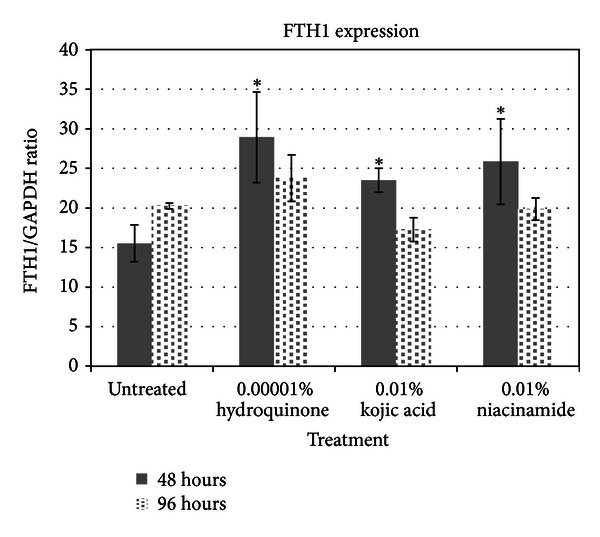
Ferritin protein expression assay results.

The expression of ferritin protein mirrored nicely the genomic data, showing upregulation by all three skin lighteners with 48 hours (Figure 6). The expression of ferritin then diminished after 96 hours for all the treatments.

Presently, the role of iron in the tanning process is relatively unexplored. Jimbow demonstrated that exposure of skin to UV light increases expression of both ferrous (Fe^+2^) and ferric (Fe^+3^) ions in melanosomes [16]. Palumbo et al. has demonstrated, however, that it appears that ferrous ions are the critical oxidative iron species that influence tyrosinase activity by upregulating the enzyme's activity [10, 11]. Ros et al. have suggested that ferrous ions may act to accelerate the hydroxylation of tyrosine which can increase the functionality of tyrosinase to form DOPA [17]. However, ferritin principally binds ferric ions, keeping such ions in a nontoxic state within the cells. Maresca et al. has demonstrated that ferritin downregulation caused melanin suppression in melanoma cells [18]. The extensive upregulation of ferritin within cells treated with skin lightening agents suggests that the cells are accumulating high levels of ferric ions at the expense, perhaps, of ferrous ions. This would be consistent with the observed reductions in melanin and suggests that the role of ferrous ions in skin lightening is presently an overlooked pathway to skin lightening. Applegate et al. has reported that ferritin expression is increased in melanocytes exposed to UVB radiation and that expression of ferritin in radiation-stressed skin cells should be considered a defensive response to oxidative stress [19]. This suggests, counterintuitively, that the skin lighteners examined here are offering a UV-type stress to the melanocytes, as measured by ferritin increase, and yet they are causing reductions in melanin production which does not happen with UV radiation. 

The influx of iron appears to occur quickly and then to subside as the levels of ferritin are high within 48 hours, but begin returning to more normal levels within 96 hours. This protein expression behavior seems to mirror pretty closely the expression of tyrosinase as well. It would seem that when tyrosinase expression is upregulated, it is important for ferritin expression to also be upregulated. This would suggest that the interplay between tyrosinase activity and ferrous ions should be explored more closely.

**Figure 7:**
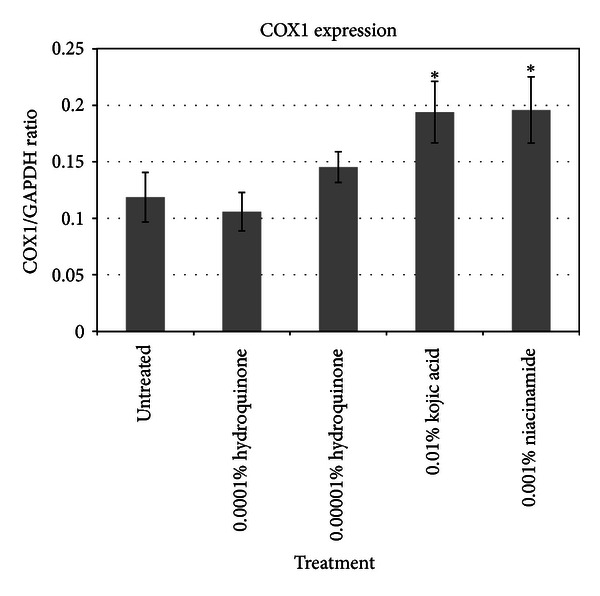
Expression of cytochrome c oxidase in treated keratinocytes.

**Figure 8:**
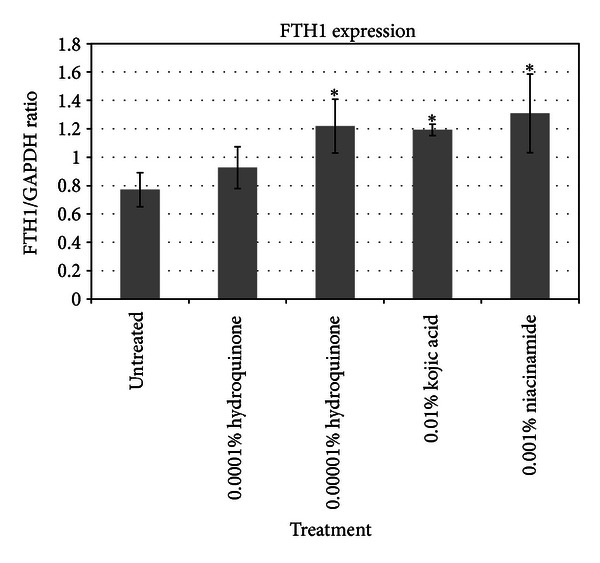
Expression of ferritin protein in treated keratinocytes.

To extend the study of the skin lighteners to additional skin cells, each skin lightener was also added to normal human epidermal keratinocytes (NHEK) at comparable concentrations as those used on melanocytes. The results of the gene expression for two key genes of interest, COX1 and FTH1, are shown in Table 2. It can be noted that comparable to the results from the melanocyte array results, there is a pronounced influence of these skin lighteners on COX1 and FTH1 expression. These results were further confirmed by protein assays as shown in Figures 7 and 8. 

What is very interesting is that all these skin lighteners also show pronounced upregulation of the ferritin gene and ferritin protein in keratinocytes indicating that in both melanocytes and keratinocytes the expression of ferric ion appears to be significant upon addition of these well established skin lighteners to both cell lines. The apparent upregulation of ferritin and cytochrome c oxidase in keratinocytes treated with skin lighteners again suggests a stress response similar to UV radiation. Applegate et al. has demonstrated that in skin cells exposed to UV radiation upregulation of ferritin is a stress response [20]. The consistency of ferritin and cytochrome c oxidase response in two cell lines exposed to well-established skin lighteners suggests that the mechanisms by which these skin lighteners function may still be vaguely understood. Nevertheless, the apparent responses indicate that the skin cells are responding to these ingredients as though they have been stressed and yet, in cell culture as well as *in vivo*, these ingredients are demonstrated to be successful skin lightening ingredients.

## Figures and Tables

**Table 1:** Summary of gene expression indicated by ratio of medians for three skin lightening agents, Hydroquinone (0.0001%), kojic acid (0.01%), and niacinamide (0.01%) looking at tyrosinase (TYR), cytochrome c oxidase-1 (COX1), melanocortin-1 receptor (MC1R), and ferritin (FTH1) expression. Ratios of medians higher than 1.3 typically indicate a statistically significant upregulation of the gene. Values less than 0.7 typically demonstrate a statistically significant downregulation of the gene.

Gene symbol	Hydroquinone	Kojic acid	Niacinamide
TYR	1.528	1.61	2.219
COX1	2.538	2.235	2.696
MC1R	0.438	0.716	0.879
FTH1	2.999	2.133	2.92

**Table 2:** Summary of gene expression indicated by ratio of medians for three skin lightening agents, hydroquinone (0.0001%), kojic acid (0.01%), and niacinamide (0.01%) looking at cytochrome c oxidase-1 (COX1) and ferritin (FTH1) expression on normal human epidermal keratinocytes. Ratios of medians higher than 1.3 typically indicate a statistically significant upregulation of the gene. Values less than 0.7 typically demonstrate a statistically significant downregulation of the gene.

Gene symbol	Hydroquinone	Kojic acid	Niacinamide
COX1	1.782	4.347	1.529
FTH1	2.478	3.295	5.896
